# Magnetic resonance spectroscopy metabolite profiles predict survival in paediatric brain tumours

**DOI:** 10.1016/j.ejca.2012.09.002

**Published:** 2013-01

**Authors:** Martin Wilson, Carole L. Cummins, Lesley MacPherson, Yu Sun, Kal Natarajan, Richard G. Grundy, Theodoros N. Arvanitis, Risto A. Kauppinen, Andrew C. Peet

**Affiliations:** a School of Cancer Sciences, University of Birmingham, Birmingham, UK; b Birmingham Children’s Hospital NHS Foundation Trust, Birmingham, UK; c Public Health, Epidemiology and Biostatistics, University of Birmingham, Birmingham, UK; d School of Electronic, Electrical & Computer Engineering, University of Birmingham, Birmingham, UK; e Medical Physics and Imaging, University Hospital Birmingham, Birmingham, UK; f Children’s Brain Tumour Research Centre, University of Nottingham, Nottingham, UK; g Clinical Research and Imaging Centre and School of Experimental Psychology, University of Bristol, Bristol, UK

**Keywords:** MRS, Metabolism, ^1^H, Proton, Lipids, Glutamine, LCModel, Grade, Non-invasive, Childhood

## Abstract

**Background:**

Brain tumours cause the highest mortality and morbidity rate of all childhood tumour groups and new methods are required to improve clinical management. ^1^H magnetic resonance spectroscopy (MRS) allows non-invasive concentration measurements of small molecules present in tumour tissue, providing clinically useful imaging biomarkers. The primary aim of this study was to investigate whether MRS detectable molecules can predict the survival of paediatric brain tumour patients.

**Patients and methods:**

Short echo time (30 ms) single voxel ^1^H MRS was performed on children attending Birmingham Children’s Hospital with a suspected brain tumour and 115 patients were included in the survival analysis. Patients were followed-up for a median period of 35 months and Cox-Regression was used to establish the prognostic value of individual MRS detectable molecules. A multivariate model of survival was also investigated to improve prognostic power.

**Results:**

Lipids and scyllo-inositol predicted poor survival whilst glutamine and N-acetyl aspartate predicted improved survival (*p* < 0.05). A multivariate model of survival based on three MRS biomarkers predicted survival with a similar accuracy to histologic grading (*p* < 5e–5). A negative correlation between lipids and glutamine was found, suggesting a functional link between these molecules.

**Conclusions:**

MRS detectable biomolecules have been identified that predict survival of paediatric brain tumour patients across a range of tumour types. The evaluation of these biomarkers in large prospective studies of specific tumour types should be undertaken. The correlation between lipids and glutamine provides new insight into paediatric brain tumour metabolism that may present novel targets for therapy.

## 1 Introduction

Of the solid tumours typically occurring in childhood, brain tumours are the most common and have the highest mortality rate.^1^ Surgery, chemotherapy and radiotherapy are commonly used in isolation or combination to treat these tumours but advances in clinical management are required to improve survival rates and reduce long-term morbidity such as effects on cognitive development.^2,3^

Diagnosis remains the most important determinant of treatment and whilst this is available from a major surgical resection in many cases, in others, a diagnosis is made from a small biopsy or on clinical and imaging appearances alone. Molecular tests on the tumour tissue are providing new prognostic markers^4,5^ and these are starting to be incorporated into clinical management strategies. Novel non-invasive biomarkers would add to this improved tumour characterisation and would have the advantage of being available for cases where surgery was not performed.

^1^H magnetic resonance spectroscopy (MRS) is a non-invasive technique that measures the concentration of variety of biomolecules from a volume of interest.^6^ The technique is widely available clinically and easily appended to a standard magnetic resonance imaging (MRI) examination, which is routinely performed at diagnosis on children with brain tumours. The two main MRS protocol choices are duration of echo time and single versus multi voxel acquisition. Short echo time MRS is the most suitable investigation for detecting the maximum amount of metabolite information, provided suitable analysis methods are used.^7,8^ Single voxel MRS is generally preferred over multi-voxel spectroscopic imaging where disease is localised since it generally provides better quality data at shorter echo times for metabolite quantification.^9^

Abnormal metabolism in tumours has been recognised for many years^10^ and this area of research continues to provide new insight into tumour biology.^11^ MRS is a powerful method for the detection of tumour metabolism *in-vivo* and metabolic profiles have been shown to characterise brain tumours non-invasively.^12,13^ Classification methods based on MRS profiles have also been shown to be effective in both adult^14^ and childhood brain tumours^15^ providing information on tumour characterisation useful for clinical management. High-resolution *in-vitro* MRS analysis of tumours has also identified a number of potentially useful metabolites that may be detectable on future clinical MR platforms.^16,17^

In addition to metabolites, MRS is effective at measuring the level of mobile lipids (MLs),^18^ which are often present at high levels in brain tumour tissue. A number of studies have shown a significant correlation between the level of MRS detectable lipids and tumour grade in adult gliomas^19–21^ and similar findings have been found in a more limited number of studies in childhood brain tumours.^22^

The primary aim of this study was to determine whether metabolite levels measured by MRS are able to predict the survival of paediatric brain tumour patients in a clinical setting. Single voxel, short echo time MRS was used to ensure quantification of both small molecular weight species and MR detectable lipids.

## 2 Patients and methods

### 2.1 Patients

All patients undergoing MR imaging at Birmingham Children’s Hospital as part of their clinical investigations for a suspected brain tumour were eligible to be enroled on this study. The accrual period was between September 2003 and July 2009 and patients were followed up until January 2010. Dates of death and progression were determined from the West Midlands tumour registry database and clinical records. Histopathologic, clinical and radiological features, as available, were used to form a diagnosis and reviewed by a multidisciplinary team. All graded tumours were biopsy proven. Ungraded tumours were either unbiopsied or biopsied and found to have a WHO diagnosis with no associated grade. Approval was obtained from the research ethics committee and informed consent given by parents/guardians.

### 2.2 MRI/MRS

MRI and MRS were carried out, prior to the patient receiving treatment, on a 1.5 T Siemens Symphony Magnetom with a single channel head coil and a 1.5 T GE Signa Excite scanner equipped with an 8-channel head coil. Standard imaging included T1 and T2 weighted images of the brain followed by gadolinium contrast administration and then T1 weighted images of the head and spine where appropriate. The conventional imaging set was used to delineate the margins of the primary tumour from known characteristics^23^ and the voxel for MRS was placed entirely within this region encompassing as much of the solid component of the lesion as possible.

Point resolved single voxel spectroscopy (PRESS)^24^ was performed with an echo time of 30 ms and a repetition time of 1500 ms. Cubic voxels of either 2 cm or 1.5 cm length were used depending on the size of the tumour. Water suppressed data were acquired with 128 repetitions from the larger voxels and 256 repetitions from the smaller ones. A corresponding water unsuppressed spectrum was also acquired with four scans for use as a concentration reference. Unprocessed MRS signals were analysed using the LCModel™ software package^7^ (version 6.2-0). Each spectrum was fitted using an experimentally acquired basis set and the SPTYPE = ‘tumour’ parameter was set for all analyses, recommended for data which may have low levels of N-Acetyl aspartate (NAA). Metabolite concentrations were scaled using the water reference acquisition, assuming an NMR-visible water molarity of (35880 mM).^25^

Each spectrum and its associated voxel placement were reviewed. Data were rejected if any of the following conditions were met: the voxel was placed closer than 4 mm to lipid containing structures, a high level of non-involved brain was within the voxel, the baseline was unstable, obvious artefacts were present, the signal-to-noise ratio (SNR) was less than 4 or the overall metabolite linewidths exceeded 0.15 ppm.

### 2.3 Statistical methods

The following metabolite lipid and macromolecular quantities were used in subsequent analyses: creatine (Cr); glutamine (Gln); glutamate (Glu); lactate (Lac); myo-inositol (m-Ins); scyllo-inositol (s-Ins); taurine (Tau); phosphocholine (PC); glycerophosphocholine (GPC); N-Acetylaspartate (NAA); N-Acetylaspartylglutamate (NAAG); lipid signals at 0.9 and 1.3 ppm and macromolecular signals at 0.9, 1.2, 1.4, 1.7 and 2.0 ppm. The following metabolite amplitudes were combined since they are difficult to resolve reliably: phosphocholine and glycerophosphocholine (TCho); and NAA and NAAG (TNAA). All quantities were divided by their standard deviation. Since individual lipid (Lip) and macromolecule signals (MM) are known to be highly correlated these signals were combined to form average quantities labelled Lip and MM respectively. Finally, each quantity was split into quartiles to prevent outliers from dominating the analysis. Whilst LCModel™ does measure other molecules, they are typically not at high enough concentration to be detected in tumour tissue and were therefore not considered in this study.

To test the primary hypothesis, univariate Cox-Regression was performed on each individual measured molecular quantity. A multivariate model of survival was also investigated using Cox-Regression and model simplification was performed using backward stepwise model selection. All statistical analysis was performed using the survival library written for the R software package.^26^

## 3 Results

**Fig. 1:**
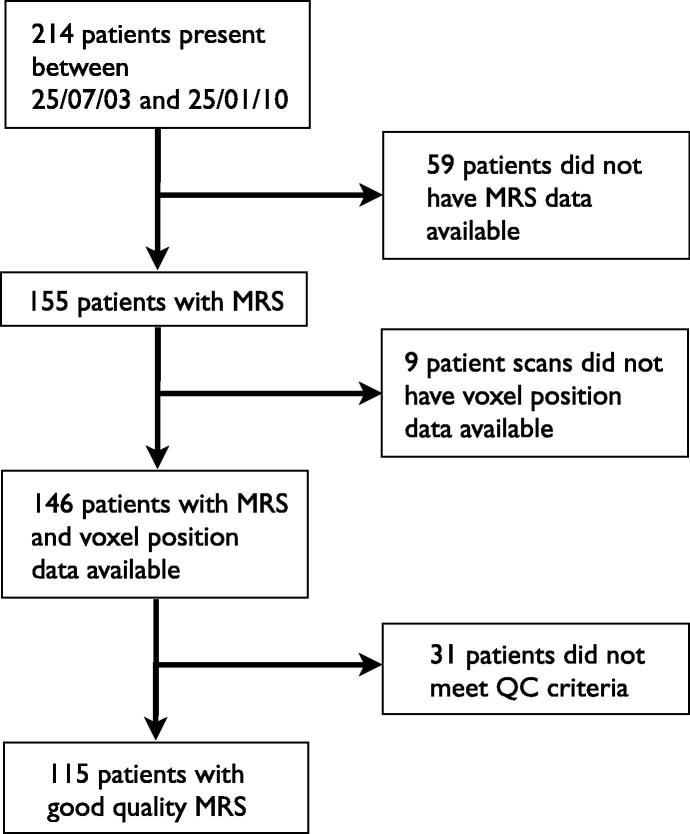
Flow diagram of patients studied.

Two hundred and fourteen patients presented with a suspected brain tumour during the accrual period and 115 were eligible for inclusion in the survival analysis. A flow chart illustrating the reasons for exclusion is shown in Fig. 1. All patient deaths were a direct result of their primary disease. The mean age of patients was 90 months with a standard deviation of 55 months and 68% were male. From the patients included in the survival analysis, 78 had graded tumours and 37 were ungraded.

**Fig. 2:**
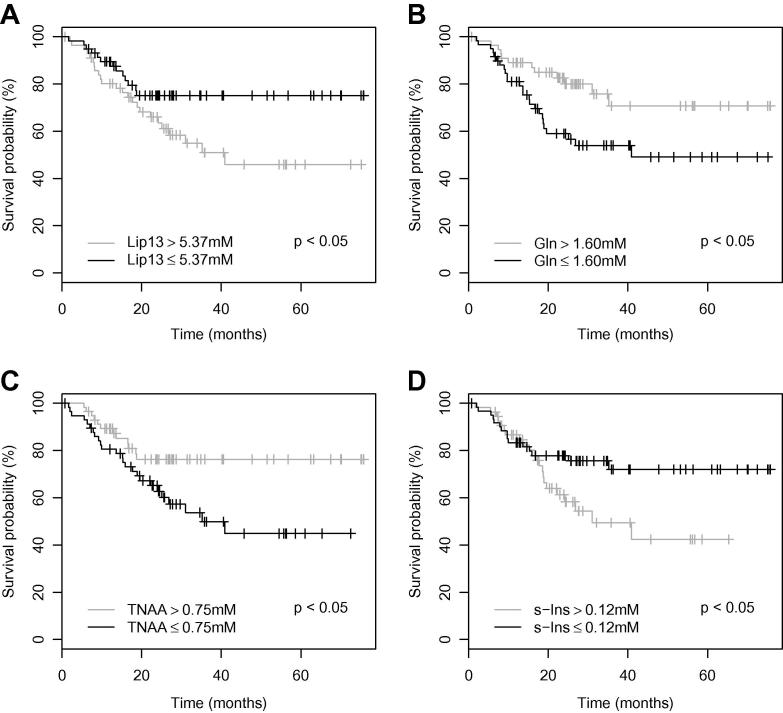
A Kaplan–Meier survival plots for (A) lipids at 1.3 PPM, (B) glutamine, (C) total NAA and (D) scyllo-inositol. Significance values represent the Chi square test for equality.

Hazard ratios, estimated by Cox-Regression, for each molecular quantity are given in Table 1. Four of the 11 quantities considered were found to be significant predictors of survival based on likelihood ratio tests (*p* < 0.05). The corresponding Kaplan–Meier survival curves are shown in Fig. 2; median values were used as cut-offs.

The average and standard deviation of all the four significant quantities are given in Table 2, grouped by tumour diagnosis. A summary of the distribution of diagnoses and frequency of deaths is also provided in Table 2. Patients diagnosed with pilocytic astrocytoma, optic pathway glioma, tectal plate glioma, DNET, germinoma or ependymoma in general had high survival rates. Conversely, patients with medulloblastoma, diffuse pontine glioma, atypical teratoid/rhabdiod tumour and glioblastoma had lower survival rates, confirming that the relative survival prospects for the more common tumour groups was in good agreement with other studies.^27^

An informative multivariate Cox-Regression model of survival is summarised in Table 3. The combination of glutamine, scyllo-inositol and lipids yielded a significant model based on a likelihood ratio test (*p* = 9e–3). The hazard ratios indicated that glutamine is a marker of improved survival whereas lipids and scyllo-inositol are markers of poor survival, consistent with Table 1.

**Fig. 3:**
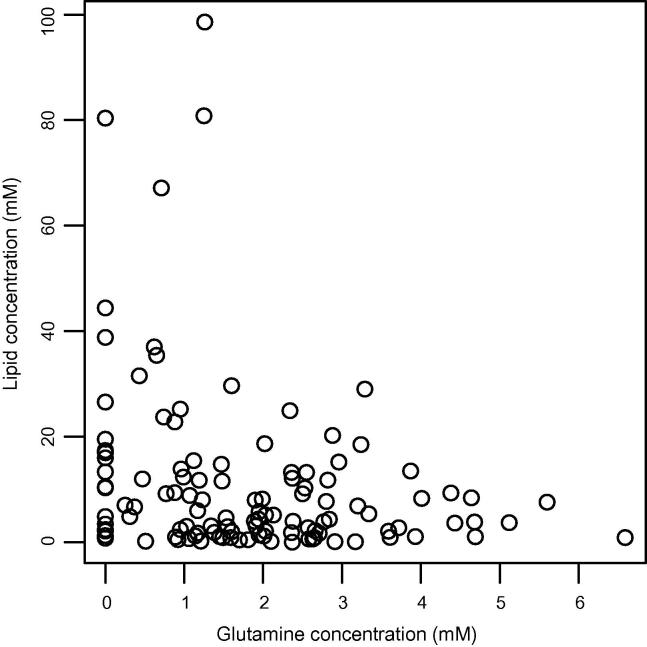
A scatterplot of glutamine and lipid concentrations.

A scatterplot of glutamine and lipid concentrations for the cohort is shown in Fig. 3. A significant negative Pearson product-moment correlation coefficient (*r* = –0.26, *p* = 0.0046) was found between these quantities indicating a possible metabolic link between these molecules.

**Fig. 4:**
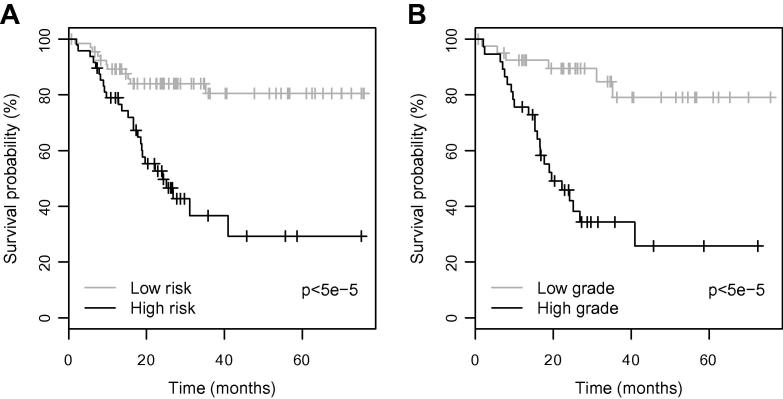
A Kaplan–Meier survival plots for (A) the risk model predicted by MRS profiles for all tumours and (B) high grade and low grade tumours. Significance values represent the Chi square test for equality.

A simple method for demonstrating how the multivariate Cox-Regression model could be used to stratify patients is described as follows. We define a risk score (*z*) as the sum of the following values:

z=0.252×Lip+0.315×s-Ins-0.263×Gln

where coefficients are derived from the hazard ratios in Table 3. A cut-off value of 0.9 was found to best discriminate between high and low risk cases where *z* > 0.9 denotes a high risk case and *z* ⩽ 0.9 denotes a low risk case. Fig. 4, part (A) shows Kaplan–Meier survival curves for the high and low risk cases. A significant difference was found between the survival prospects of the two groups using the Chi square test for equality (*p* < 5e–5). Part (B) shows Kaplan–Meier survival curves for high grade (WHO III, IV) and low grade (WHO I, II) tumours for comparison. Similar predictive accuracies between the presented survival model and histopathological grade are apparent from the survival curves.

**Fig. 5:**
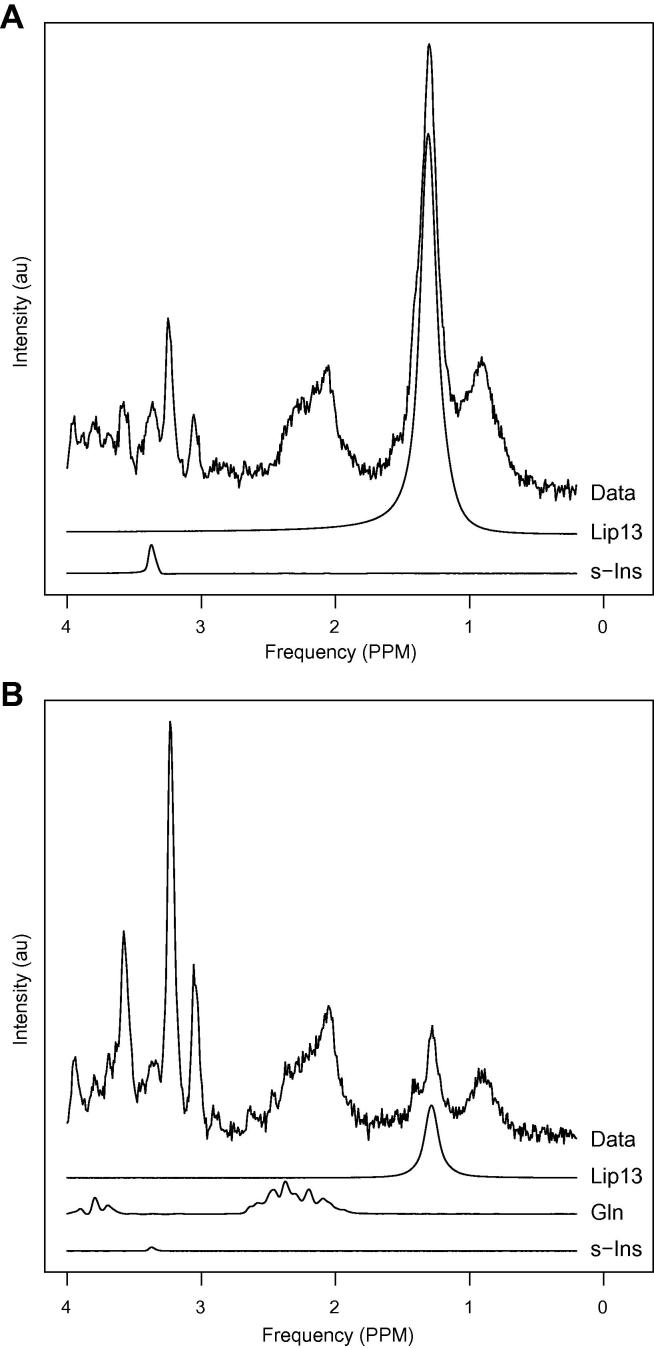
Example spectra with (A) high-risk and (B) low-risk features. Relevant fitted metabolite signals are shown below the spectral data. Gln has not been plotted in part (A) since it had an estimated concentration of zero.

Example spectra are shown in Fig. 5 to illustrate the spectral appearance of the fitted signals found to be important in the Cox-Regression. Both spectra were taken from patients diagnosed with classic medulloblastoma. The spectra from patients (A) and (B) exhibit features of high-risk and low-risk disease respectively. Patient (B) was alive at the end of the study (31 months) whereas patient (A) died after 9 months. The risk scores (*z*) for the high-risk and low-risk cases were 2.0 and 0.3 respectively.

## 4 Discussion

This study has shown that the metabolite and lipid levels of tumours, detected non-invasively by short echo time MRS at diagnosis, predict survival in a cohort of children with brain tumours. Our results confirm that tumour lipids, previously reported as a prognostic biomarker,^28^ are robust in this larger cohort. Furthermore, due to the shorter echo time (30 ms) used in this study, we were able to identify glutamine and scyllo-inositol as useful prognostic markers.

Our study found that the combination of lipids, glutamine and scyllo-inositol were able to predict survival with a similar accuracy to histologic grade (Fig. 4). Most paediatric brain tumours undergo surgery; therefore a histologic grading is often available. However, MRS provides additional prognostic information on the tumour, which may strengthen, or challenge that obtained from histopathology and other tests. Where a tumour associated with a favourable prognosis has an MRS profile implying a more aggressive course, treatment intensification may be considered. For tumours that are not biopsied, approximately a quarter of all cases, prognostic information is still available from MRS since it is a non-invasive technique. Where this information contradicts that available from other methods, such as conventional imaging, an argument could be made for biopsying the tumour or altering the treatment plan. In addition, tissue biopsy may present a risk of morbidity for tumours in certain locations and MRS can provide important non-invasive information for these cases.

Many children with brain tumours are treated on sophisticated protocols which stratify patients on an increasingly complex set of prognostic markers combining clinical information, conventional imaging, histological markers and more recently tumour biology. The role of MRS within this process needs to be determined by its inclusion in large multi-centre clinical trials and this study demonstrates the importance of including MRS in such trials.

A strong association between MRS detectable lipids and tumour grade has been reported in adult gliomas^19–21^ and childhood brain tumours^22^ suggesting that high intracellular lipid levels are a non-invasive marker of brain tumour malignancy. Work on cultured cells, tumour tissue *ex-vivo* and animal models have shown that an increase in MRS detectable lipids is associated with cell stress,^29^ apoptosis^30^ and hypoxia as a result of compromised vascularity.^31^ MRS detectable lipids are therefore associated with several factors known to be present in aggressive tumours that have a poor prognosis.

The link between lipids and malignancy is expected since one of the key requirements for rapidly proliferating cells is a boost in lipid synthesis, providing constituent molecules for cellular membranes. The recent work of Metallo et al.^32^ has shown that glutamine, rather than glucose, is the major carbon source for lipid synthesis in A549 adenocarcinoma cells under hypoxic conditions through a previously underappreciated pathway. In our work, intra-cellular glutamine has been shown to be a prognostic marker of survival in paediatric brain tumours, supporting evidence of its importance to tumour proliferation. Furthermore, an inverse correlation between intra-cellular glutamine and mobile lipids has been shown (Fig. 3) supporting recent studies linking glutamine and lipogenesis.^33^ Fig. 5 illustrates the variance of glutamine and lipids present in medulloblastoma, and further work is warranted to investigate therapeutic targeting of these pathways.

The MRS measurement of glutamate and glutamine is difficult at 1.5 T, due to their overlap with other signals. However, two recent studies comparing high-resolution *in-vitro* and *in-vivo* MRS^34,35^ have shown a good correlation between the techniques for these metabolites, suggesting that their prognostic strength is high enough to outweigh the inaccuracy in their measurement. Further *in-vivo* MRS studies using higher field strengths, such as 3 T which are now commonly available in clinics,^36^ optimised acquisition parameters^37^ and advanced analysis methods^8^ will improve the detection accuracy of these and other metabolites in clinical practice.

In addition to lipids and glutamine, N-acetyl aspartate and scyllo-inositol were found to be significant prognostic markers. N-acetyl aspartate is commonly used as a marker of neuronal density and viability and has been noted in a previous study as a potential prognostic marker when combined with choline.^28^ The exact function of N-acetyl aspartate in tumours is unknown. In diffuse tumours it may indicate the level of entrapped neurones, however it is thought to play an important role in osmoregulation and this could be relevant to its presence in tumour cells.^38^ Scyllo-inositol is a novel prognostic marker, however its role in tumour metabolism is poorly understood and warrants further study.

In conclusion, the study has demonstrated that short echo time single voxel MRS can be used to predict patient survival in paediatric brain tumours with a similar accuracy to histologic grading. A novel link between intracellular glutamine and mobile lipids has been identified, a pertinent finding given recent evidence that glutamine is crucial to lipogenesis in hypoxic tumour cells. These findings provide information that may improve both the clinical management and molecular understanding of paediatric brain tumours.

## Conflict of interest statement

None declared.

## Figures and Tables

**Table 1:** A summary of univariate survival hazard ratios and significance values for all MRS signals analysed. The likelihood ratio test was used for significance testing.

Signal	Hazard ratio	*p*
Cr	1.023	0.874
Gln	0.713	0.022
Glu	1.057	0.710
Lac	1.001	0.992
Lip	1.373	0.039
m-Ins	1.067	0.661
MM	0.838	0.228
s-Ins	1.354	0.050
Tau	1.231	0.153
TCho	1.024	0.875
TNAA	0.734	0.047

**Table 2:** Patients by diagnostic groups with mean (SD) quantities (derived from concentration quartiles) of MRS signals related to survival.

Diagnosis	Frequency	Events	Gln	Lip	s-Ins	TNAA
Astrocytoma high grade	7	5	2.4 (1.1)	3.0 (1.2)	1.7 (1.3)	1.9 (0.9)
Astrocytoma low grade	31	3	2.7 (1.1)	2.4 (1.0)	1.7 (0.6)	2.6 (1.0)
ATRT	3	3	2.0 (1.7)	3.7 (0.6)	2.3 (1.5)	1.0 (0.0)
Biopsied other	5	2	2.2 (1.3)	2.8 (1.3)	2.8 (1.3)	1.6 (0.9)
DNET	5	0	2.6 (0.9)	1.4 (0.5)	2.8 (1.1)	3.6 (0.9)
Ependymoma	7	3	3.1 (1.1)	3.0 (1.2)	2.7 (1.4)	2.3 (1.0)
Germ cell	6	0	2.3 (1.5)	3.5 (0.5)	2.8 (0.8)	2.3 (1.2)
PNET	24	13	2.0 (1.1)	3.2 (0.6)	2.7 (1.2)	1.6 (0.7)
Unbiopsied diffuse pontine glioma	9	8	1.9 (0.9)	1.6 (0.7)	3.3 (0.9)	3.8 (0.4)
Unbiopsied optic pathway glioma	9	0	3.1 (0.8)	1.2 (0.4)	2.6 (0.9)	3.4 (0.5)
Unbiopsied other	9	0	2.7 (1.2)	1.4 (1.0)	2.9 (1.2)	3.4 (0.7)

**Table 3:** A summary of the multivariate Cox Regression model for all tumours; based on MRS detectable metabolites.

Signal	Hazard ratio	Lower 95% conf.	Upper 95% conf.	*p*
Glutamine	0.769	0.568	1.042	0.090
Scyllo-inositol	1.370	1.014	1.851	0.041
Lipid	1.268	0.939	1.761	0.116

Likelihood ratio test *p* = 0.0091, 3 df.
